# High expression of miR-25 predicts favorable chemotherapy outcome in patients with acute myeloid leukemia

**DOI:** 10.1186/s12935-019-0843-9

**Published:** 2019-05-07

**Authors:** Mingshan Niu, Yuan Feng, Ninghan Zhang, Tingting Shao, Huihui Zhang, Rong Wang, Yao Yao, Ruosi Yao, Qingyun Wu, Jiang Cao, Xuejiao Liu, Yubo Liu, Kailin Xu

**Affiliations:** 1 Blood Diseases Institute, Affiliated Hospital of Xuzhou Medical University, Xuzhou Medical University, Xuzhou, Jiangsu China; 2 Department of Hematology, Affiliated Hospital of Xuzhou Medical University, Xuzhou, Jiangsu China; 3 Institute of Nervous System Diseases, Xuzhou Medical University, Xuzhou, Jiangsu China; 4 School of Life Science & Medicine, Dalian University of Technology, Panjin, China

**Keywords:** Acute myeloid leukemia, miR-25, Clinical outcome, Chemotherapy, Allo-HSCT

## Abstract

**Background:**

Acute myeloid leukemia (AML) pertains to a hematologic malignancy with heterogeneous therapeutic responses. Improvements in risk stratification in AML patients are warranted. MicroRNAs have been associated with the pathogenesis of AML.

**Methods:**

To examine the prognostic value of miR-25, 162 cases with de novo AML were classified into two groups according to different treatment regimens.

**Results:**

In the chemotherapy group, cases with upregulated miR-25 expression showed relatively longer overall survival (OS; *P* = 0.0086) and event-free survival (EFS; *P* = 0.019). Multivariable analyses revealed that miR-25 upregulation is an independent predictor for extended OS (HR = 0.556, *P* = 0.015) and EFS (HR = 0.598, *P* = 0.03). In addition, allogeneic hematopoietic stem cell transplantation (allo-HSCT) circumvented the poor prognosis that was related to miR-25 downregulation with chemotherapy. The expression level pattern of miR-25 coincided with AML differentiation and proliferation, which included HOXA and HOXB cluster members, as well as the HOX cofactor MEIS1. The MYH9 gene was identified as a direct target of miR-25.

**Conclusions:**

The miR-25 levels are correlated with prognosis in AML independently of other powerful molecular markers. The expression of miR-25 may contribute to the selection of the optimal treatment regimen between chemotherapy and allo-HCST for AML patients.

## Background

Acute myeloid leukemia (AML) is a group of clonal malignant diseases that derive from the hematopoietic stem cells. AML is characterized by a large group of germinal cells, which leads to a loss of normal hematopoietic function [1]. The clinical prognosis of patients with AML is various. The differences in outcomes among AML patients depend on multiple intrinsic factors [2, 3]. With the development of methodologies of massive sequencing, it has been demonstrated that somatic mutations in NPM1, FLT3, CEBPA, IDH1 and IDH2 are connected to prognosis in AML [4]. To be specific, patients with mutated FLT3 have a dismal outcome, while mutations in NPM1 and CEBPA are related with favorable prognosis. The advent of chemotherapy and allogeneic hematopoietic stem cell transplantation (allo-HSCT) has significantly improved AML treatment outcomes [5]. Relapse and refractory of leukemia remain the most disturbing problems in AML patients [6]. Thus, it is urge to explore more reliable and effective prognostic biomarkers to enhance the capacity of prediction and thus improve the outcome of AML by choosing optimal therapeutic approach.

MicroRNAs are short non-coding RNAs, which are implicated in a diverse group of critical cellular mechanisms, such as apoptosis, differentiation, cell cycle progression, and immune responses [7]. Recently, more and more attentions have been focused on the prognostic role of microRNAs in AML. A recent study has shown that the upregulation of miR-181a facilitates better survival of AML patients who are cytogenetically normal [8]. However, AML patients who are cytogenetically normal and upregulated miR-212 and miR-3151 have shorter overall and disease-free survival [9, 10]. However, most microRNA analyses did not differentiate the AML patients treated with chemotherapy and allo-HSCT. Thus, microRNAs may have varied prognostic roles in chemotherapy and allo-HSCT treatment group, respectively.

MiR-25, a member of miR-106b-25 cluster, is located on human chromosome 7q22.1 [11]. Previous studies revealed that miR-25 was involved in many kinds of cancers [12]. It has been identified that miR-25 is a potential biomarker for pediatric AML based on Pipeline of Outlier MicroRNA Analysis (POMA) model [13]. More important, Garzon et al. [14] reported that miR-25 is significantly down-regulated in 122 newly diagnosed AML samples compared with CD34^+^ normal cells. However, clinical and prognostic role of miR-25 in AML are still unclear. A total of 162 recently diagnosed de novo AML patients were enrolled in this evaluation. The cases were placed into two groups based on the treatment that they received. The present study suggested that miR-25 is a solitary AML prognostic biomarker. Furthermore, our study revealed that allo-HSCT would be more beneficial to patients showing downregulated miR-25.

## Patients and methods

### Patients

Approximately 162 patients with a diagnosis of de novo AML were included in this study. The data sets used in this investigation were acquired from The Cancer Genome Atlas (TCGA). A single-institution tissue banking strategy endorsed by the human studies committee of Washington University was used in this study. All of the patients provided their written informed consent. AML diagnosis and classification were made according to the French–American–British (FAB) and the World Health Organization (WHO) criteria. The cases were placed into two groups according to the clinical treatment received. Ninety patients accepted chemotherapy, and the rest accepted allo-HSCT.

### Gene-expression profiling

The samples from 155 patients both had been obtained mRNA and microRNA expression data. These data were applied to identify the mRNA-expression signature associated with miR-25 expression. The sequencing read count for each miRNA was normalized to Reads per million reads (RPM). The mRNA expression values were logged (base 2) prior to analysis [15]. Spearman correlation was used to correlate the mRNA-expression profile with miR-25 expression. Hierarchical clustering analysis was used to reorder the gene rows. To screen for target genes of miR-25, Targetscan, miRNApath and miRDB website tools were implemented. Gene Ontology enrichment assessment of genes in miR-340 related signature was performed with the Database for Annotation, Visualization, and Integrated Discovery (DAVID).

### Statistical analysis

The clinical endpoints of this investigation on treatment outcomes included overall survival (OS) and event-free survival (EFS). OS pertains to the time interval from diagnosis to death or last follow-up of the patient. EFS is described as the time interval from diagnosis to disease progression, relapse, or death attributed to any cause. The patients were assigned to the high or low expression groups based on the median miR-25 expression. Descriptive statistics (median and/or range) were used to summarize patients’ clinical and molecular characteristics. To elucidate the role of miR-25 expression in AML clinical and molecular features, the Pearson Chi-square and Fisher’s exact tests were used to screen for significant differences between two categorical variables. In addition, the Mann–Whitney’s *U* test was used for continuous variables. For univariable and multivariable analysis, a Cox proportional hazards model was employed to determine the effect of various risk factors on patient OS and EFS. The limited backward elimination procedure was applied to assess hazard ratios (HRs) and *P* values. Kaplan–Meier analysis was performed to determine the impact of miR-25 expression on OS and EFS. Statistical analysis was conducted with SPSS and GraphPad Prism. Differences among variables were determined to be statistically significant when the *P* value was < 0.05.

## Results

### Correlation analysis of miR-25 expression and clinical characteristics

To establish the correlation among miR-25 expression and various clinical profiles, we assigned the patients who underwent chemotherapy and allo-HSCT to one of two groups according to median miR-25 expression levels, respectively. The associations of the clinical features with miR-25 expression levels are summarized in Table 1. In the chemotherapy group, subjects who exhibited upregulated miR-25 had a higher percentage of RUNX1-RUNX1T1 compared to those with downregulated expression (*P* = 0.026). In addition, high miR-25 expresser involved in more good risk cases of AML (*P* = 0.002). However, no significant differences were observed in gender, age, WBC count, BM blast, PB blast, FAB subtypes, FLT3-ITD, NPM1, DNMT3A, RUNX1, MLL-PTD, TP53, IDH1 and IDH2 among the high and low miR-25 expression group. In the allo-HSCT group, study participants with upregulated miR-25 exhibited a lower frequency for FLT3-ITD mutations (*P* = 0.045) compared to those with downregulated miR-25. No significant differences in as far as gender, age, WBC count, BM blast, PB blast, and mutations in the NPM1, RUNX1, DNMT3A, MLL-PTD, IDH1, IDH2, and TP53 genes were observed among the upregulated and downregulated miR-25 groups.

**Table 1:** Comparison of clinical and molecular characteristics with miR-25 expression in patients with AML

Characteristic	Chemotherapy group			Allo-HSCT group		
	High miR-25 (n = 45)	Low miR-25 (n = 45)	*P*	High miR-25 (n = 36)	Low miR-25 (n = 36)	*P*
Age/years, median	61.4 (22–82)	64.4 (31–88)	0.49	47.3 (22–72)	49.4 (18–69)	0.535
Age group/n (%) (years)			0.495			0.793
< 60	16 (35.6)	12 (26.7)		27 (75)	25 (69.4)	
≥ 60	29 (64.4)	33 (73.3)		9 (25)	11 (30.6)	
Gender/n (%)			0.289			0.634
Male	22 (48.9)	28 (62.2)		22 (61.1)	19 (52.8)	
Female	23 (51.1)	17 (37.8)		14 (38.9)	17 (47.2)	
WBC/× 109/L, median	32.4 (0.7–297.4)	51.8 (1.5–298.4)	0.059	36.0 (0.6–223.8)	39.8 (1.2–118.8)	0.248
BM blast/%, median	69 (32–99)	67.2 (30–92)	0.955	66.2 (34–99)	70.2 (30–100)	0.277
PB blast/%, median	39.5 (0–98)	35.1 (0–97)	0.320	46.1 (0–96)	48.9 (0–94)	0.752
FAB subtypes/n (%)						
M0	4 (8.9)	4 (8.9)	1.000	6 (16.7)	3 (8.3)	0.478
M1	13 (28.9)	7 (15.6)	0.204	7 (19.4)	16 (44.4)	0.042
M2	12 (26.7)	9 (20)	0.619	12 (33.3)	7 (19.4)	0.285
M4	11 (24.4)	13 (28.9)	0.812	8 (22.2)	6 (16.7)	0.767
M5	4 (8.9)	9 (20)	0.230	2 (5.6)	2 (5.6)	1.000
M6	1 (2.2)	0 (0.0)	1.000	0 (0.0)	1 (2.8)	1.000
M7	0 (0.0)	2 (4.4)	0.494	1 (2.8)	0 (0.0)	1.000
No date	0 (0.0)	1 (2.2)	1.000	0 (0.0)	1 (2.8)	1.000
Karyotype/n (%)						
Normal	18 (40)	26 (57.8)	0.140	15 (41.7)	19 (52.7)	0.479
Complex	5 (11.1)	7 (15.6)	0.758	6 (16.6)	6 (16.6)	1.000
Poor	0 (0.0)	5 (11.1)	0.056	1 (2.8)	4 (11.1)	0.357
Intermediate	8 (17.8)	2 (4.4)	0.090	6 (16.7)	3 (8.4)	0.478
MLL	1 (2.2)	2 (4.4)	1.000	3 (8.3)	0 (0.0)	0.239
CBFβ-MYH11	6 (13.3)	1 (2.2)	0.110	4 (11.1)	1 (2.8)	0.357
BCR-ABL1	0 (0.0)	1 (2.2)	1.000	0 (0.0)	2 (5.6)	0.493
RUNX1-RUNX1T1	6 (13.3)	0 (0.0)	0.026	1 (2.8)	0 (0.0)	1.000
N.D.	1 (2.2)	1 (2.2)	1.000	0 (0.0)	1 (2.8)	1.000
Risk(cyto)/n (%)						
Good	12 (26.7)	1 (2.2)	0.002	5 (13.9)	1 (2.8)	0.199
Intermediate	26 (57.8)	29 (64.4)	0.665	21 (58.3)	20 (55.5)	1.000
Poor	6 (13.3)	14 (31.1)	0.074	10 (27.8)	14 (38.9)	0.454
Other	1 (2.2)	1 (2.2)	1.000	0 (0.0)	1 (2.8)	1.000
FLT3-ITD/n (%)			0.784			0.045
Presence	9 (20.0)	7 (15.6)		4 (11.1)	12 (33.3)	
Absence	36 (80.0)	38 (84.4)		32 (88.9)	24 (66.7)	
NPM1/n (%)			0.175			0.064
Presence	11 (24.4)	18 (40)		6 (16.7)	14 (38.9)	
Absence	34 (75.6)	27 (60)		36 (83.3)	22 (61.1)	
DNMT3A/n (%)			0.157			1.000
Presence	9 (20)	16 (35.6)		9 (25)	9 (25)	
Absence	36 (80)	29 (64.6)		27 (75)	27 (75)	
RUNX1/n (%)			0.714			0.710
Presence	5 (11.1)	3 (6.7)		5 (13.9)	3 (8.3)	
Absence	40 (89.9)	42 (93.3)		31 (86.1)	33 (91.7)	
MLL-PTD/n (%)			1.000			
Presence	2 (4.4)	3 (6.7)		2 (5.6)	2 (5.6)	
Absence	43 (95.6)	42 (93.3)		34 (94.4)	34 (94.4)	
TP53/n (%)			0.522			1.000
Mutation	4 (8.9)	7 (15.6)		2 (5.6)	2 (5.6)	
Wild type	41 (91.1)	38 (84.4)		34 (94.4)	34 (94.4)	
CEBPA/n (%)			1.000			0.055
Mutation	1 (2.2)	2 (4.4)		7 (19.4)	1 (2.8)	
Wild type	44 (97.8)	43 (95.6)		29 (80.6)	35 (97.2)	
IDH1/n (%)			1.000			0.514
Mutation	3 (6.7)	4 (8.9)		4 (11.1)	7 (19.4)	
Wild type	42 (93.3)	41 (91.1)		32 (88.9)	29 (80.6)	
IDH2/n (%)			1.000			0.260
Mutation	5 (11.1)	4 (8.9)		2 (5.6)	6 (16.7)	
Wild type	40 (88.9)	41 (91.1)		34 (94.4)	30 (83.3)	

Mann–Whitney test was used for continuous variables. Chi square tests were used for categorical variables

*WBC* white blood cell, *BM* bone marrow, *PB* peripheral blood, *FAB* French–American–British classification

### Prognostic value of miR-25 profiles in AML patients

**Fig. 1:**
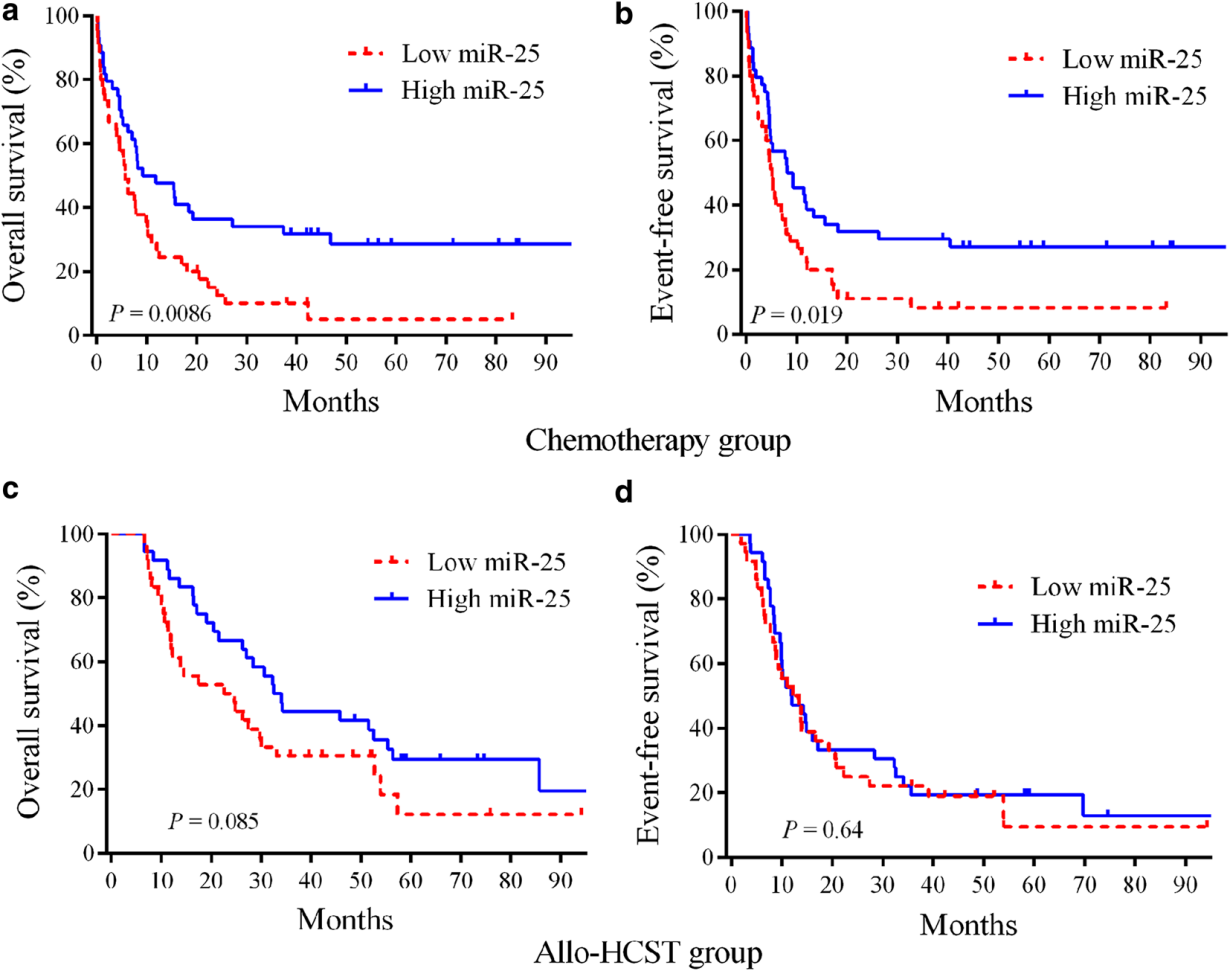
Kaplan–Meier survival curves of AML patients stratified based on miR-25 expression. **a**, **b** In the chemotherapy group, the high miR-25 expressers had significantly prolonged OS and EFS (n = 90) compared with low miR-25 expressers. **c**, **d** There were no significant differences in patients undergoing allo-HSCT between high and low miR-25 groups (n = 72)

We performed Kaplan–Meier analysis and log-rank test to assess the prognostic value of miR-25 profiles in AML patients. The chemotherapy group showed that AML patients with upregulated miR-25 were connected to better EFS (*P* = 0.019) and OS (*P* = 0.0086) relative to those with downregulated miR-25 (Fig. 1a, b). However, AML patients who received allo-HSCT did not exhibit any connection among prognosis and miR-25 expression (Fig. 1c, d). These findings revealed that miR-25 may be utilized as a chemotherapy-specific prognostic marker for AML.

### High level of miR-25 is independently associated with favorable prognosis

To determine whether miR-25 expression could be used as an independent predictor for AML patient survival, we conducted univariate and multivariate Cox analyses. For the chemotherapy group, univariate analysis revealed that the upregulation of miR-25 was connected with longer EFS (HR = 0.598, 95% CI 0.376–0.951, *P* = 0.030) and OS (HR = 0.556, 95% CI 0.347–0.890, *P* = 0.015). Furthermore, multivariate cox analysis indicated that miR-25 upregulation was independently connected with longer EFS (HR = 0.561, 95% CI 0.333–0.943, *P* = 0.029) and OS (HR = 0.502, 95% CI 0.296–0.851, *P* = 0.011) after adjustment of mutation status for the FLT3-ITD, NPM1, DNMT3A, RUNX1, IDH1, and IDH2 genes and WBC count (Table 2).

**Table 2:** Univariate and multivariate analyses in patients treated with chemotherapy

Variables	EFS		OS	
	HR (95% CI)	*P*-value	HR (95% CI)	*P*-value
Univariate analyses				
MiR-25 (high vs low)	0.598 (0.376–0.951)	0.030	0.556 (0.347–0.890)	0.015
WBC (< 20 vs ≥ 20 × 109/L)	0.939 (0.594–1.484)	0.786	0.936 (0.591–1.484)	0.779
FLT3-ITD (positive vs negative)	1.242 (0.693–2.224)	0.467	1.192 (0.665–2.136)	0.555
NPM1 (mutated vs wild)	1.168 (0.721–1.893)	0.527	1.044 (0.640–1.704)	0.862
DNMT3A (mutated vs wild)	1.491 (0.909–2.446)	0.114	1.432 (0.868–2.362)	0.160
RUNX1 (mutated vs wild)	1.464 (0.700–3.064)	0.312	1.591 (0.759–3.335)	0.219
ITDH1 (mutated vs wild)	1.043 (0.452–2.405)	0.922	0.908 (0.366–2.254)	0.836
ITDH2 (mutated vs wild)	0.981 (0.487–1.977)	0.956	0.991 (0.492–1.995)	0.979
Multivariate analyses				
MiR-25 (high vs low)	0.561 (0.333–0.943)	0.029	0.502 (0.296–0.851)	0.011
WBC (< 20 vs ≥ 20 × 109/L)	0.884 (0.537–1.456)	0.629	0.927 (0.563–1.527)	0.766
FLT3-ITD (positive vs negative)	1.489 (0.778–2.848)	0.229	1.578 (0.815–3.054)	0.176
NPM1 (mutated vs wild)	0.877 (0.476–1.615)	0.674	0.760 (0.411–1.405)	0.382
DNMT3A (mutated vs wild)	1.421 (0.787–2.568)	0.244	1.416 (0.787–2.550)	0.246
RUNX1 (mutated vs wild)	1.730 (0.768–3.897)	0.186	1.805 (0.805–4.050)	0.152
ITDH1 (mutated vs wild)	1.141 (0.448–2.904)	0.782	1.074 (0.397–2.906)	0.889
ITDH2 (mutated vs wild)	1.039 (0.480–2.251)	0.922	1.042 (0.483–2.248)	0.916

*EFS* event-free survival, *OS* overall survival, *WBC* white blood cell

Univariate analysis of the allo-HSCT group suggested that AML cases harboring FLT3-ITD mutations had shorter EFS (HR = 1.873, 95% CI 1.020–3.437, *P* = 0.043) and OS (HR = 1.998, 95% CI 1.053–3.788, *P* = 0.034). Patients with mutations only in the RUNX1 gene exhibited shorter OS (HR = 2.253, 95% CI 1.046–4.849, *P* = 0.038). Multivariate analysis indicated that FLT3-ITD and RUNX1 remained independent outcome predictors after adjusting for all other prognostic factors (Table 3). However, allo-HSCT patients did not show any significant differences between upregulated and downregulated miR-25 expression.

**Table 3:** Univariate and multivariate analyses in patients treated with allo-HSCT

Variables	EFS		OS	
	HR (95% CI)	*P*-value	HR (95% CI)	*P*-value
Univariate analyses				
MiR-25 (high vs low)	0.886 (0.553–1.473)	0.641	0.625 (0.364–1.073)	0.088
WBC (< 20 vs ≥ 20 × 109/L)	1.530 (0.910–2.571)	0.108	0.949 (0.554–1.628)	0.851
FLT3-ITD (positive vs negative)	1.873 (1.020–3.437)	0.043	1.998 (1.053–3.788)	0.034
NPM1 (mutated vs wild)	0.913 (0.515–1.619)	0.755	0.879 (0.478–1.617)	0.678
DNMT3A (mutated vs wild)	1.106 (0.615–1.989)	0.737	1.269 (0.686–2.347)	0.447
RUNX1 (mutated vs wild)	1.375 (0.650–2.907)	0.404	2.253 (1.046–4.849)	0.038
ITDH1 (mutated vs wild)	0.985 (0.498–1.949)	0.966	0.810 (0.382–1.718)	0.582
ITDH2 (mutated vs wild)	0.569 (0.227–1.425)	0.229	0.931 (0.368–2.357)	0.880
Multivariate analyses				
MiR-25 (high vs low)	0.788 (0.421–1.476)	0.457	0.510 (0.266–0.978)	0.043
WBC (< 20 vs ≥ 20 × 109/L)	1.343 (0.756–2.386)	0.314	0.827 (0.450–1.519)	0.540
FLT3-ITD (positive vs negative)	2.222 (1.044–4.729)	0.038	2.201 (0.951–5.096)	0.065
NPM1 (mutated vs wild)	0.586 (0.280–1.227)	0.156	0.560 (0.249–1.259)	0.161
DNMT3A (mutated vs wild)	1.058 (0.549–2.037)	0.867	1.514 (0.774–2.963)	0.226
RUNX1 (mutated vs wild)	1.483 (0.620–3.545)	0.376	2.671 (1.114–6.402)	0.028
ITDH1 (mutated vs wild)	1.265 (0.535–2.944)	0.592	0.781 (0.305–1.999)	0.606
ITDH2 (mutated vs wild)	0.524 (0.183–1.498)	0.228	0.499 (0.175–1.424)	0.194

*EFS* event-free survival, *OS* overall survival, *WBC* white blood cell

### Allo-HSCT may circumvent poor patient outcomes that are related to downregulated miR-25 expression

**Fig. 2:**
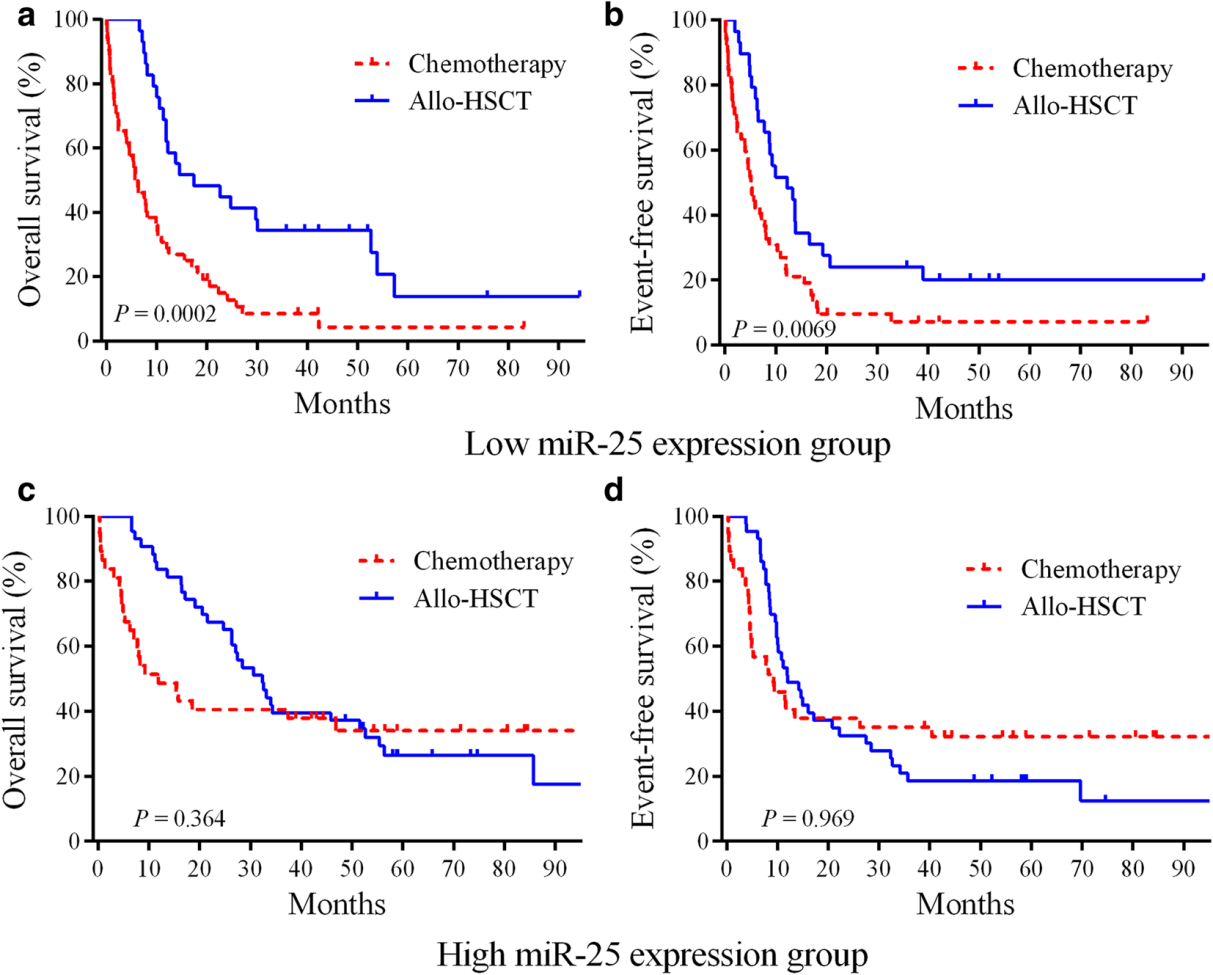
Allo-HSCT treatment circumvents the unfavorable outcomes of AML patients showing downregulated miR-25 expression. **a**, **b** A total of 162 cases were placed into two groups according to the median miR-25 expression levels. In the downregulated miR-25 group, the Kaplan–Meier survival curves of AML patients classified based on chemotherapy (n = 52) and allo-HSCT (n = 29) treatment. **c**, **d** In the upregulated miR-25 group, the Kaplan–Meier survival curves of AML patients classified based on chemotherapy (n = 38) and allo-HSCT (n = 43) treatment

To determine whether allo-HSCT therapy could circumvent the severe prognosis that was associated with downregulated miR-25, the whole cohort of 162 cases was split into two groups according to the median miR-25 expression levels. In the downregulated miR-25 group, the AML cases who received allo-HSCT showed significantly longer EFS (HR = 0.515, 95% CI 0.327–0.831, *P* = 0.0069) and OS (HR = 0.405, 95% CI 0.250–0.639, *P* = 0.0002) relative to cases who underwent standard chemotherapy alone (Fig. 2a, b). For the upregulated miR-25 group, no obvious differences in EFS (*P* = 0.969) and OS (*P* =0.364) were observed among the allo-HSCT and chemotherapy regimens. Thus, the AML patients showing downregulated miR-25 may benefit from treatment with allo-HSCT.

### Biological insights into miR-25 profiles in AML

**Fig. 3:**
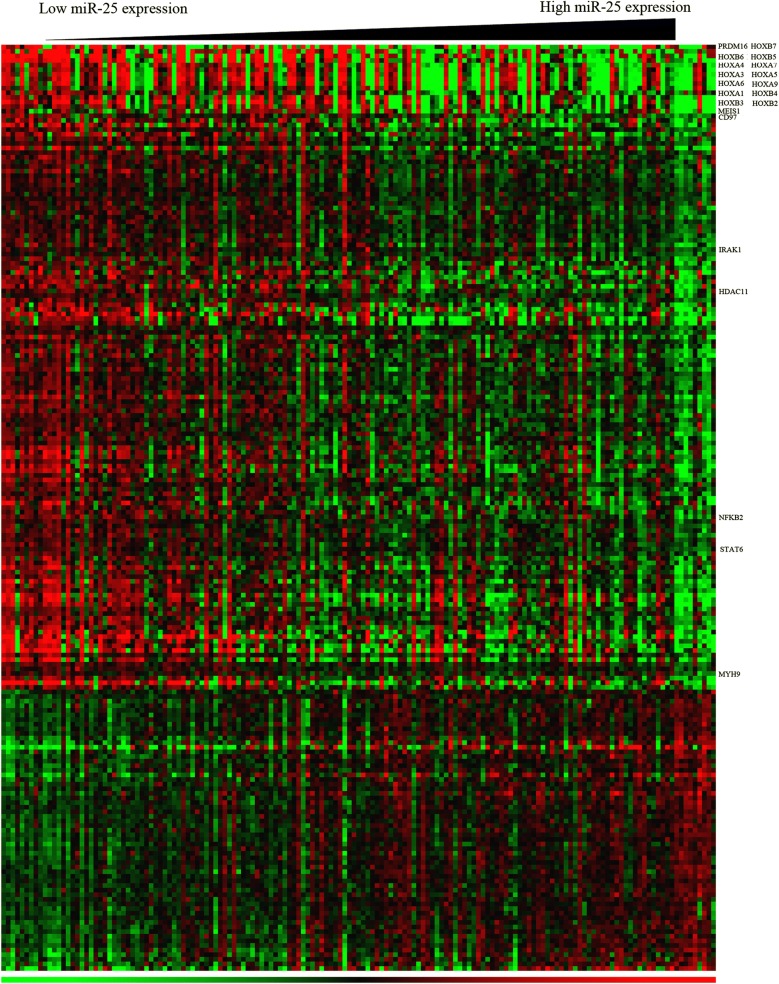
Heat map of miR-25-associated gene-expression signature in patients with AML. The columns represent patients and the rows represent genes. The columns are ordered from left to right according to increasing expression levels of miR-25. The hierarchical cluster analysis was performed to order rows. The expression levels of various genes are represented by nodes of different colors, ranging from the lowest (green) to the highest (red)

To generate insights into the molecular mechanism of miR-25, we analyzed a gene expression signature that was connected with miR-25 expression among AML cases. An association between the expression of 205 genes and miR-25 was observed. Among these genes, 145 were negatively correlated and 60 were positively correlated with the expression of miR-25 (Fig. 3). MiR-25 expression was inversely correlated with the expression of HOXA and HOXB, as well as the HOX cofactor MEIS1. Notably, these genes are crucial for the leukemogenesis and self-renewal capacities of AML [8, 16, 17]. Furthermore, we discovered that the expression of miR-25 was negatively connected with the levels of the PRDM16, Which involved in AML translocation [18]; CD97, an EGF-TM7 receptor [19]; IRAK1, which activates NF-κB pathways by the interaction with TRAF6 [20]; NFKB2, a pro-inflammatory response gene [21]; MYH9, which predicts unfavorable outcome of AML [22]; HDAC11, a epigenetic regulator. Notably, MYH9 was a in silico predicted target of miR-25.

Gene Ontology showed that genes involved in cellular metabolic process, system development, immune system process, transcription, hematopoietic or lymphoid organ development, hemopoiesis and myeloid cell differentiation were markedly overrepresented among differentially expressed genes associated with miR-25 expression (Table 4).

**Table 4:** Gene ontology terms of biological processes in the miR-25 associated expression profile

GO ID	GO terms	Percentage of members of the GO term present in the miR-25 profile	*P*-value FDR
GO:0031323	Regulation of cellular metabolic process	46.9	0.029
GO:0048522	Regulation of cellular process	40.3	0.029
GO:0048731	System development	37.2	0.039
GO:0010604	Regulation of macromolecule metabolic process	28.5	0.013
GO:0002376	Immune system process	26.5	0.004
GO:0045893	Regulation of transcription	16.8	0.037
GO:0048534	Hematopoietic or lymphoid organ development	14.7	< 0.001
GO:0002520	Immune system development	14.7	< 0.001
GO:0030097	Hemopoiesis	13.7	< 0.001
GO:0001501	Skeletal system development	9.6	0.012
GO:0030099	Myeloid cell differentiation	8.1	0.009

*GO* Gene Ontology

## Discussion

AML has been considered to occur as the result of genetic abnormalities, including chromosomal rearrangements, gene deregulations and mutations [23]. The deregulated expression of microRNAs in AML can influence cell proliferation, survival and hematopoietic differentiation [24]. The association of microRNAs with prognosis in heterogeneous patients with AML is still largely unclear. In this evaluation, the upregulated of miR-25 was determined to be an independently favorable prognosticator of AML cases who were administered chemotherapy. Furthermore, allo-HSCT may overcome the poor prognosis of AML cases with low miR-25 expression.

A correlation between aberrant miRNA expression and AML prognosis has been established [25, 26]. However, most of previous microRNA markers is restricted to AML without cytogenetic abnormalities. In our study, univariate and multivariate analyses demonstrated that miR-25 is an independently biomarker for cases administered chemotherapy. High miR-25 expression can predict favorable outcome. The prognostic role of miR-25 is different with previously established prognostic factors in a heterogeneous population of AML. MiR-25, as an independent outcome predictor, may improve the current clinical risk-based classification of patients with AML.

To further understand the biological insight into the molecular mechanism underlying miR-25, we identified genes significantly correlated with miR-25 expression. We discovered that the expression of miR-25 negatively connected with the levels of PRDM16, HOXAs, HOXBs, MEIS1, CD97, IRAK1, NFKB2 and MYH9. HOXA and HOXB gene clusters are the common characters of AML [27, 28]. Of these genes, HOXB4 is positively involved in the renewal of hematopoietic stem cell [29, 30]. A previous study has shown that HOXA9 contributes to the proliferation, apoptosis, and differentiation processes of leukemia [31]. In addition, HOXA9 has been correlated with poor AML prognosis [32]. Prior evaluations have revealed that IRAK1 may be utilized as a therapeutic target for AML, and TRAF6 may be used to activate pathways such as NFKB, MAPK, and AKT [20, 33]. PRDM16, also known as MEL1, is highly homologous to MDS1/EVI1. High expression of PRDM16 can predict the adverse outcome of AML [18]. Moreover, MYH9 has also been predicted as a direct target of miR-25. High expression of MYH9 can induce resistant to chemotherapy and predict poor clinical outcome in AML [22]. Taken together, the miR-25-associated gene-expression profiling analyses provide insights into the leukemogenic role of genes that are either direct or indirect targets of miR-25. Therefore, the miR-25-associated gene-expression signature analysis give novel insights into the oncogenic role of these genes. These miR-25-related genes could contribute to the chemotherapeutic responses of AML patients.

The FMS-like tyrosine kinase 3 (FLT3) gene is pivotal to hematopoietic stem cell proliferation and differentiation [34]. FLT3 mutations take a great account of most frequent genetic aberrations in AML [35]. FLT3-ITD mutation is one of FLT3 mutations, which can keep the tyrosine kinase persistently active, and result in the abnormal proliferation of leukemic cells. Mutations in the FLT-ITD gene have been associated with higher risk for relapse and poor OS and EFS [36]. Consistent with the conclusion, our data suggested that FLT3-ITD mutation is a poor outcome marker in patients undergoing allo-HSCT. These analysis results indicate that allo-HSCT cannot overcome all adverse prognosis of molecular markers. The findings of this study have revealed that allo-HSCT circumvents the poor chemotherapy outcomes that are related to downregulated miR-25 expression. Thus, low miR-25 expression may be employed as a predictor of adverse prognoses among patients who received chemotherapy, as well as identify patients who require strategies in selecting the best treatment regimen, i.e., chemotherapy and/or allo-HCST.

## Conclusion

In conclusion, high expression of miR-25 was identified to independently predict favorable survival in a highly heterogeneous population of patients with AML. Our findings may offer more information for the therapeutic strategies and the prediction of patients with AML, which may improve the survival and reduce the relapse of them. More importantly, allo-HSCT circumvents poor chemotherapeutic outcomes in cases with downregulated miR-25. The expression levels of miR-25 may thus be utilized in determining whether chemotherapy or allo-HSCT is the optimal treatment regimen for a specific AML patient.

## Data Availability

The datasets supporting the conclusions of this article are included within this article. Clinical data for all patients, including the treatment approach and outcomes data, are publicly accessible from the TCGA website (https://cancergenome.nih.gov).

## Abbreviations

AML: acute myeloid leukemia
TCGA: The Cancer Genome Atlas
WHO: World Health Organization
Allo-HSCT: allogeneic hematopoietic stem cell transplantation
OS: overall survival
EFS: event-free survival

## References

[1] Ding L, Ley TJ, Larson DE, Miller CA, Koboldt DC, Welch JS (2012). Clonal evolution in relapsed acute myeloid leukaemia revealed by whole-genome sequencing. Nature.

[2] Papaemmanuil E, Gerstung M, Bullinger L, Gaidzik VI, Paschka P, Roberts ND (2016). Genomic classification and prognosis in acute myeloid leukemia. N Engl J Med.

[3] Niu M, Shen Y, Qi J, Liu X, Sang W, Wu Q (2017). Effects of realgar (As_4_S_4_) on degradation of PML-RARA harboring acquired arsenic-resistance mutations. Ann Hematol.

[4] Bullinger L, Dohner K, Dohner H (2017). Genomics of acute myeloid leukemia diagnosis and pathways. J Clin Oncol.

[5] Chen X, Xie H, Wood BL, Walter RB, Pagel JM, Becker PS (2015). Relation of clinical response and minimal residual disease and their prognostic impact on outcome in acute myeloid leukemia. J Clin Oncol.

[6] Khwaja A, Bjorkholm M, Gale RE, Levine RL, Jordan CT, Ehninger G (2016). Acute myeloid leukaemia. Nat Rev Dis Primers.

[7] Nowek K, Sun SM, Dijkstra MK, Bullinger L, Dohner H, Erkeland SJ (2016). Expression of a passenger miR-9* predicts favorable outcome in adults with acute myeloid leukemia less than 60 years of age. Leukemia.

[8] Schwind S, Maharry K, Radmacher MD, Mrozek K, Holland KB, Margeson D (2010). Prognostic significance of expression of a single microRNA, miR-181a, in cytogenetically normal acute myeloid leukemia: a Cancer and Leukemia Group B study. J Clin Oncol.

[9] Sun SM, Rockova V, Bullinger L, Dijkstra MK, Dohner H, Lowenberg B (2013). The prognostic relevance of miR-212 expression with survival in cytogenetically and molecularly heterogeneous AML. Leukemia.

[10] Eisfeld AK, Marcucci G, Maharry K, Schwind S, Radmacher MD, Nicolet D (2012). miR-3151 interplays with its host gene BAALC and independently affects outcome of patients with cytogenetically normal acute myeloid leukemia. Blood.

[11] Qu J, Li M, Zhong W, Hu C (2015). Prognostic role of microRNA-25 in cancers: evidence from a meta-analysis. Int J Clin Exp Med.

[12] Wang M, Yang YO, Jin Q, Shang L, Zhang J (2018). Function of miR-25 in the invasion and metastasis of esophageal squamous carcinoma cells and bioinformatical analysis of the miR-106b-25 cluster. Exp Ther Med.

[13] Yan W, Xu L, Sun Z, Lin Y, Zhang W, Chen J (2015). MicroRNA biomarker identification for pediatric acute myeloid leukemia based on a novel bioinformatics model. Oncotarget.

[14] Garzon R, Volinia S, Liu CG, Fernandez-Cymering C, Palumbo T, Pichiorri F (2008). MicroRNA signatures associated with cytogenetics and prognosis in acute myeloid leukemia. Blood.

[15] Zhang H, Zhang N, Wang R, Shao T, Feng Y, Yao Y (2019). High expression of miR-363 predicts poor prognosis and guides treatment selection in acute myeloid leukemia. J Transl Med.

[16] Argiropoulos B, Humphries RK (2007). Hox genes in hematopoiesis and leukemogenesis. Oncogene.

[17] Eklund EA (2007). The role of HOX genes in malignant myeloid disease. Curr Opin Hematol.

[18] Yamato G, Yamaguchi H, Handa H, Shiba N, Kawamura M, Wakita S (2017). Clinical features and prognostic impact of PRDM16 expression in adult acute myeloid leukemia. Genes Chromosomes Cancer.

[19] Wobus M, Bornhauser M, Jacobi A, Krater M, Otto O, Ortlepp C (2015). Association of the EGF-TM7 receptor CD97 expression with FLT3-ITD in acute myeloid leukemia. Oncotarget.

[20] Beverly LJ, Starczynowski DT (2014). IRAK1: oncotarget in MDS and AML. Oncotarget.

[21] Morikawa M, Nakano S, Mitsui N, Murasawa H, Masuki S, Nose H (2017). Effects of dried tofu supplementation during interval walking training on the methylation of the NFKB2 gene in the whole blood of older women. J Physiol Sci.

[22] Yu M, Wang J, Zhu Z, Hu C, Ma Q, Li X (2017). Prognostic impact of MYH9 expression on patients with acute myeloid leukemia. Oncotarget.

[23] Grimwade D, Ivey A, Huntly BJ (2016). Molecular landscape of acute myeloid leukemia in younger adults and its clinical relevance. Blood.

[24] Dell’Aversana C, Giorgio C, D’Amato L, Lania G, Matarese F, Saeed S (2018). miR-194-5p/BCLAF1 deregulation in AML tumorigenesis. Leukemia.

[25] Shivarov V, Dolnik A, Lang KM, Kronke J, Kuchenbauer F, Paschka P (2016). MicroRNA expression-based outcome prediction in acute myeloid leukemia: novel insights through cross-platform integrative analyses. Haematologica.

[26] Yang C, Shao T, Zhang H, Zhang N, Shi X, Liu X (2018). MiR-425 expression profiling in acute myeloid leukemia might guide the treatment choice between allogeneic transplantation and chemotherapy. J Transl Med.

[27] Spencer DH, Young MA, Lamprecht TL, Helton NM, Fulton R, O’Laughlin M (2015). Epigenomic analysis of the HOX gene loci reveals mechanisms that may control canonical expression patterns in AML and normal hematopoietic cells. Leukemia.

[28] Kontro M, Kumar A, Majumder MM, Eldfors S, Parsons A, Pemovska T (2017). HOX gene expression predicts response to BCL-2 inhibition in acute myeloid leukemia. Leukemia.

[29] Antonchuk J, Sauvageau G, Humphries RK (2002). HOXB4-induced expansion of adult hematopoietic stem cells ex vivo. Cell.

[30] Miyake N, Brun AC, Magnusson M, Miyake K, Scadden DT, Karlsson S (2006). HOXB4-induced self-renewal of hematopoietic stem cells is significantly enhanced by p21 deficiency. Stem Cells.

[31] Collins C, Wang J, Miao H, Bronstein J, Nawer H, Xu T (2014). C/EBPalpha is an essential collaborator in Hoxa9/Meis1-mediated leukemogenesis. Proc Natl Acad Sci USA.

[32] Collins CT, Hess JL (2016). Role of HOXA9 in leukemia: dysregulation, cofactors and essential targets. Oncogene.

[33] Rhyasen GW, Bolanos L, Fang J, Jerez A, Wunderlich M, Rigolino C (2013). Targeting IRAK1 as a therapeutic approach for myelodysplastic syndrome. Cancer Cell.

[34] Shlush LI, Zandi S, Mitchell A, Chen WC, Brandwein JM, Gupta V (2014). Identification of pre-leukaemic haematopoietic stem cells in acute leukaemia. Nature.

[35] Stone RM, Mandrekar SJ, Sanford BL, Laumann K, Geyer S, Bloomfield CD (2017). Midostaurin plus chemotherapy for acute myeloid leukemia with a FLT3 mutation. N Engl J Med.

[36] Brunet S, Labopin M, Esteve J, Cornelissen J, Socie G, Iori AP (2012). Impact of FLT3 internal tandem duplication on the outcome of related and unrelated hematopoietic transplantation for adult acute myeloid leukemia in first remission: a retrospective analysis. J Clin Oncol.

